# The quantity and quality of B-cell immunity against SARS-CoV-2 in children with cancer and hematological diseases

**DOI:** 10.3389/fimmu.2025.1613778

**Published:** 2025-07-02

**Authors:** Eva Tiselius, Emil Sundberg, Amanda Ramilo Amor, Hanna Andersson, Renate Varnaite, Linda Kolstad, Dario Akaberi, Jiaxin Ling, Arja Harila, Shanie Saghafian-Hedengren, Tove Hoffman, Anna Nilsson

**Affiliations:** ^1^ Department of Women’s and Children’s Health, Division of Pediatric Oncology and Pediatric Surgery, Karolinska Institutet, Stockholm, Sweden; ^2^ Department of Women’s and Children’s Health, Uppsala University, Uppsala, Sweden; ^3^ Mabtech AB, Nacka Strand, Sweden; ^4^ Zoonosis Science Center (ZSC), Department of Medical Biochemistry and Microbiology, Uppsala University, Uppsala, Sweden; ^5^ Department of Children’s Oncology and Hematology, Uppsala University Hospital, Uppsala, Sweden

**Keywords:** SARS-CoV-2 infection, antibody responses, memory B cells, childhood cancer, hematological disease

## Abstract

**Background:**

Our understanding of protective immunity after natural viral infections in children with cancer and hematological diseases is restricted. Current cancer treatments cause significant immunosuppression, affecting both innate and adaptive immunity which leads to reduced B-cell and antibody responses. The aim of this study was to characterize SARS-CoV-2 immune response in children with cancer or hematological disease.

**Methods:**

A single-center study was conducted from June 2020 to June 2023, including 135 patients and 14 healthy siblings. Blood samples were obtained for serological analysis and cell-based assays. SARS-CoV-2 IgG and IgA responses were quantified using suspension multiplex immunoassay (SMIA) and enzyme-linked immunosorbent assay (IgG ELISA) while neutralizing antibody (nAb) responses were assessed by plaque reduction neutralization tests (PRNT). The memory B-cell (MBC) population was evaluated through flow cytometry and MBC responses through FluoroSpot, respectively.

**Results:**

In total, 78 patients seroconverted in response to SARS-Co-V-2 but neither immunosuppression nor cancer diagnosis significantly affected seroconversion. SARS-CoV-2 IgG and IgA levels correlated positively with increasing age, and IgA seroconversion was significantly associated with the presence of nAbs. Antigen-specific MBC responses against both spike and receptor-binding domain (RBD) were elevated in older children, while children on immunosuppression had significantly lower RBD IgG-secreting cells.

**Conclusion:**

Our results show that most pediatric oncological and hematological patients can mount a broad antibody response upon SARS-CoV-2 natural infection or vaccination, although there is a variability in their responses influenced by increasing age. MBC responses in children with immunosuppression were blunted with fewer RBD IgG-secreting cells. Essentially, our findings underscore that young children with severe treatment-related immunosuppression are at risk for less effective B-cell responses upon viral infection.

## Introduction

To date, our knowledge regarding protective immunity after viral infections in children with cancer is limited, yet children with cancer frequently suffer from viral infections during therapy. The emergence of the severe acute respiratory syndrome coronavirus 2 (SARS-CoV-2) in 2020 sparked tremendous efforts to gain new insight into the interplay between humoral and cellular immunity to achieve immune control of SARS-CoV-2 in both children and adults. Early reports indicated that children experienced more favorable outcomes than adults upon infection, in addition to lower antibody and cellular responses compared to adults upon acute infection (1, 2). In the oncological setting, the clinical outcomes of SARS-CoV-2 infections in children with cancer were also reported to be less severe compared to adult cancer patients (3–7).

Modern treatment of most childhood cancers involves multimodal therapies consisting of chemotherapy, surgery, and/or irradiation with the addition of immunotherapy and autologous or allogeneic hematological stem-cell transplantation (auto-HSCT or allo-HSCT) in selected cases. It is widely established that cancer treatment causes a high degree of immunosuppression, affecting both the innate and adaptive immune systems. Immune recovery post-treatment has been shown to take several months to years (8, 9), with several studies indicating that humoral immunity and B cells are particularly vulnerable to treatment-related immunosuppression (8–10). This, in turn, leads to reduced B-cell and antibody responses to infection and vaccination as well as the loss of previously acquired immunity, all in all resulting in an increased vulnerability to infections (9, 11, 12).

In the first years of life, the immune system matures rapidly, influencing both the quantity and quality of the humoral immune response in children (13, 14). In response to a variety of exposures, the memory B-cell (MBC) pool is established with long-lived plasma cells mainly residing in the bone marrow, ensuring long-term protection (13, 15, 16). Neutralizing antibodies (nAbs) in children are of particular interest given their role in inhibiting viral cell entry, replication, and spread to other cells (17). In SARS-CoV-2 infection, nAbs primarily target the viral receptor-binding domain (RBD) of the viral spike (S) glycoprotein and thus interfere with viral binding to the angiotensin-converting enzyme 2 (ACE-2) receptor on the host cell. Children have previously been shown to seroconvert after both symptomatic and asymptomatic SARS-CoV-2 infection and to develop nAbs with similar or even longer durability compared with adults (18–20). Vaccination with a SARS-CoV-2 mRNA vaccine in children undergoing active cancer treatment has been shown to result in both cellular and humoral immune responses with long-lasting (> one year) strong antibody responses (21, 22).

To understand if the quantity and quality of the humoral response to SARS-CoV-2 infection was influenced by patient- and/or treatment-related factors in children with cancer or hematological disease, we performed an in-depth characterization of SARS-CoV-2 infection and immune response in pediatric oncology and hematology patients. We initiated a single-center longitudinal study where patients were included and followed from June 2020 to June 2023 with repeated sampling for SARS-CoV-2 seroconversion and immunological analysis (7). Within the scope of this study, we performed an extensive serological evaluation of SARS-CoV-2 immune responses in singular individual samples from 135 patients and 14 healthy siblings, including analyses of antibodies against various SARS-CoV-2 variants, production of nAbs and assessment of MBC responses to SARS-CoV-2.

## Materials and methods

### Study design and participants

**Figure 1:**
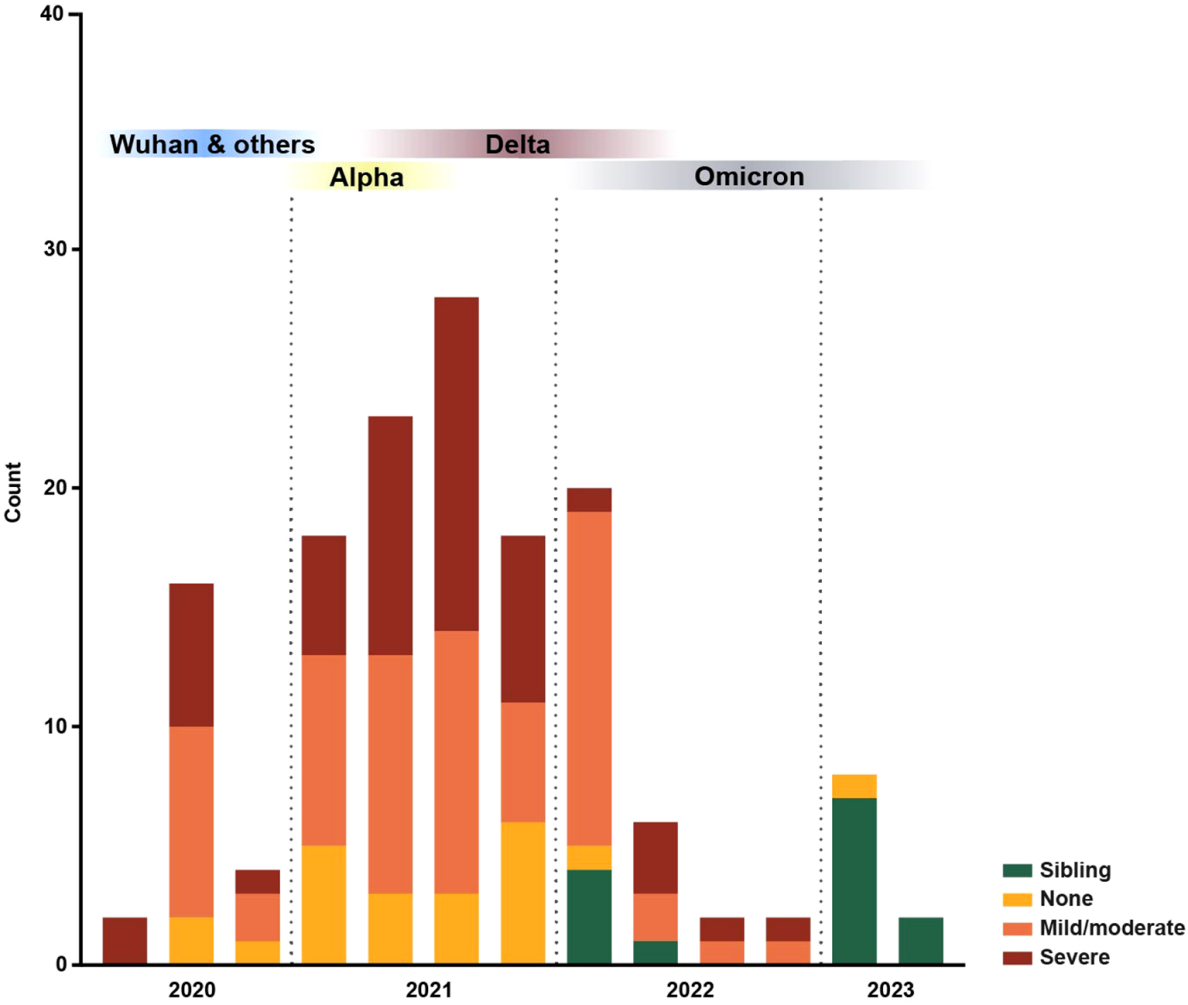
Distribution of blood samples collected over time. All patient and sibling blood samples (n = 149) collected within this study over time, color-coded by immunosuppression degree. Blood sample collection started in 2020 and ran until 2023; each bar represents a quarter. The predominant SARS-CoV-2 variants in Sweden, Wuhan and others, Alpha, Delta and Omicron, are represented based on data from the Public Health Agency of Sweden (54).

The present study was conducted as part of a prospective, longitudinal cohort study, previously described in Sundberg et al. (7). Children with cancer or hematological diseases receiving treatment or follow-up care in Uppsala, Sweden, as well as their healthy siblings, were included (**Figure 1**). The Swedish Ethical Review Authority granted ethical approval (2020–02154, 2020–04672, 2022-02068-02). The study cohort consisted of the first seropositive [Immunoglobulin (Ig) G+] blood sample from patients who seroconverted (n = 78), a matched seronegative (IgG-) blood sample within a similar time frame from patients who did not seroconvert (n = 57), as well as blood samples from healthy siblings (n = 14), resulting in 149 unique blood samples from 149 children. Of note, no children within the current study experienced severe symptoms of COVID-19. Neither PCR testing nor vaccination were performed for study purposes and study participation was independent of previous infections and SARS-CoV-2 vaccination.

### Collection of clinical data

Clinical patient data were collected through medical records, and sibling data were collected through a brief questionnaire. The degree of immunosuppression at the time of blood sampling was assessed by two pediatric oncologists using set criteria previously published (23). Four patients were IgG+ within two weeks after starting treatment which may suggest that they had been infected by SARS-CoV-2 before diagnosis.

### Collection of blood samples and total immunoglobulin analysis

Peripheral venous blood was sampled from patients in conjunction with previously planned clinical interventions, while blood sampling of siblings occurred at scheduled appointments. For the collection of serum, blood samples were collected in CAT Serum Clot Activator VACUETTE tubes (Greiner Bio-One Gmbh, Austria) and centrifuged before serum was aliquoted and stored in ultra-low temperature freezers. All serum samples were analyzed for total (i.e., not SARS-CoV-2-specific) IgG, IgA, and IgM at the local hospital laboratory (Department of Clinical Chemistry, Uppsala Academic Hospital, Uppsala, Sweden) using a Cobas Pro c503 (Roche, Switzerland).

For isolation of peripheral blood mononuclear cells (PBMCs), peripheral venous blood was collected in CPT™ Mononuclear Preparation Tubes with sodium heparin (BD Biosciences, Franklin Lakes, NJ, USA), according to the manufacturer´s instructions. Briefly, PBMCs were separated through centrifugation, washed twice with phosphate-buffered saline (PBS; Gibco, Invitrogen, Carlsbad, Ca, USA) before resuspension in freezing medium (10% dimethyl sulfoxide (Invitrogen, Carlsbad, Ca, USA) in fetal bovine serum (Sigma-Aldrich, St Louis, MO, USA)) for cryopreservation in liquid nitrogen. Due to sampling limitations, PBMCs were not obtained from all patients at all time-points.

### SARS-CoV-2 antibody quantification through a suspension multiplex immunoassay

Serum samples were analyzed using a coronavirus disease 2019 (COVID-19) suspension multiplex immunoassay (SMIA) to detect anti-SARS-CoV-2 IgG and IgA (24). Briefly, recombinant wildtype SARS-CoV-2 S protein subunit 1 (S1) (#40591-V08H, Sino Biological, Beijing, China) was coupled to magnetic beads (MagPlex microspheres; Luminex Corp., DiaSorin, Austin, Texas, USA). Diluted serum samples and conjugated beads were mixed in a 96-well microtiter plate (#650101, Greiner bio-one, Cytvia, Marlborough, MA, USA), incubated, and washed. Subsequently, biotinylated protein G (0.5 mg/mL) (#29988, Pierce Biotechnology, Thermo Fisher Scientific, Waltham, MA, USA) or biotinylated anti-human IgA (2 μg/mL) (#A18785, Invitrogen, Thermo Fisher Scientific, Waltham, MA, USA) was added followed by additional incubation and washing. Lastly, streptavidin-phycoerythrin (2 μg/mL) (#SA10044, Invitrogen, Thermo Fisher Scientific, Waltham, MA, USA) was added and the plate was incubated and washed before being analyzed in a MagPix instrument (Luminex Corp., DiaSorin, Austin, Texas, USA), measuring the median fluorescence intensity (MFI) to determine anti-S1 antibody presence. For IgG determination, an MFI ≥ 300 was classified as IgG+ and for IgA determination, an MFI ≥ 750 was classified as IgA+. The COVID-19 SMIA was similarly performed on all samples using recombinant S1 from the Delta and Omicron variants (#40591-V08H23, #40591-V08H41, Sino Biological, Beijing, China). All Delta IgG+ and Omicron IgG+, except for one Omicron IgG+, were wild-type IgG+. Therefore, wild-type IgG seropositivity was used to define seropositivity in this study and wild-type IgG MFI was used for statistical comparisons. IgG+ samples were analyzed twice and the mean value was used in the analyses.

### Enzyme-linked immunosorbent assay for confirmation of SARS-CoV-2 IgG

In addition to MFI IgG serostatus, human IgG antibodies against SARS-CoV-2 S protein (trimer) were also quantified in serum samples through a solid‐phase sandwich Enzyme‐Linked Immunosorbent Assay (ELISA) Kit (#BMS2325, Thermo Fischer Scientific, Waltham, MA, USA), according to the manufacturer’s protocol. Absorbance was measured at 450 nm using a FLUOstar Omega multimode microplate reader (BMG Labtech, Ortenberg, Germany). For quantification in seropositive samples, IgG concentrations (Units/mL) were calculated based on a standard curve generated from serial dilutions of the High Control. Values above the standard curve were further diluted and the optical density (OD) values still out of range at dilution 1:2,000 (n = 10) were assigned the highest concentration x2. To confirm seronegativity, IgG serostatus was determined by performing the qualitative method recommended by the manufacturer.

### Plaque reduction neutralization test for the quantification of neutralizing antibody responses

For determination of antibody neutralizing capability, a plaque reduction neutralization test (PRNT) was performed as previously described (25, 26). Briefly, Vero E6 cells were seeded in 24-well plates and incubated overnight (ON). On the day of analysis, serum samples were heat inactivated in 56°C for 30 minutes and diluted in Dulbecco’s Modified Eagle Medium (DMEM) (#41966029, Gibco, Thermo Fisher Scientific, Waltham, MA, USA) supplemented with Fetal Bovine Serum (FBS) (#10500064, Gibco, Thermo Fisher Scientific, Waltham, MA, USA) and penicillin-streptomycin (#PA333, Sigma-Aldrich, Merck, Darmstadt, Germany). Diluted serum samples and SARS-CoV-2 (within 5^th^ passage in Vero E6 cells, isolated in December 2020 from a Swedish patient (27)) were mixed, resulting in a final serum dilution of 1:10 and 1:20 and a virus dilution giving approximately 50 plaque-forming units (PFU) per 0.1 mL. The mixtures were incubated for 1 hour (h) and then used to inoculate the Vero E6 cells. Infected cell controls were inoculated with a similar DMEM-virus mixture, but lacking serum, while uninfected cell controls were mock-inoculated with only DMEM mixture. The serum samples were analyzed in duplicates while the virus control was analyzed in triplicates on each plate. Subsequently, the cells were overlayed with a 500 μL of a 1:1 mix of 1.6% noble agar solution in water (#A5431, Sigma-Aldrich, Merck, Darmstadt, Germany) and 2X Minimal Essential Medium (MEM) (#11935046, Gibco, Thermo Fisher Scientific, Waltham, MA, USA), supplemented with FBS and penicillin-streptomycin. After a 72-h incubation, the cells were stained with 0.5 mL of a 3% (v/v) Neutral red solution obtained diluting a stock solution (#N2889, Sigma-Aldrich, Merck, Darmstadt, Germany) in PBS or a 0.2% Crystal violet (#61135, Sigma-Aldrich, Merck, Darmstadt, Germany) in 4% paraformaldehyde solution (#100496, Sigma-Aldrich, Merck, Darmstadt, Germany), followed by an incubation for 4 h or ON, respectively. After stain removal, the mean number of plaques was counted, and the percentage of plaque reduction was calculated. Serum samples that inhibited plaque formation by ≥ 80% were considered positive for SARS-CoV-2 nAbs.

### Immunophenotyping through flow cytometry

PBMCs were washed and incubated with a dead cell marker (#L34957, Fixable Dead Cell Stain kit, Invitrogen, Carlsbad, CA, USA). After washing, the cells were once again stained with fluorescent-conjugated antibodies against several surface antigens, including CD14, CD3, and CD19 (BD Biosciences, Franklin Lakes, NJ, USA). Bulk B cells were defined by gating live CD14^–^CD3^-^CD19^+^ single cells. Data were acquired using the Novocyte 3000 (ACEA Biosciences, Santa Clara, CA, USA) and analyzed with FlowJo software (v. 10.10, FlowJo LLC, Ashland, OR, USA).

### Determination of SARS-CoV-2-specific memory B- and T-cell responses through FluoroSpot

The number of SARS-CoV-2-specific MBCs was determined with the FluoroSpot Path SARS-CoV-2 (S+RBD) Human IgG kit (#FSP-05E-RS1-1, Mabtech AB, Nacka Strand, Sweden), according to the manufacturer’s instructions. In brief, 10^6^ PBMCs/mL were stimulated with 1 mg/mL R848 and 10 ng/mL IL-2 for 72 hrs. Subsequently, 10^5^ cells were transferred to wells for total IgG analysis and 3.5x10^5^ cells into wells for antigen-specific analysis and incubated ON (37°C, 5% CO_2_). Plates were then developed according to the manufacturer’s instructions. The method was validated with medium-only wells as blanks, PBMCs from pre-pandemic samples of healthy adults as negative controls, and PBMCs from SARS-CoV-2-immunized healthy adults as positive controls (data not shown). RBD and S spot-forming unit (SFU) counts were normalized to the total IgG SFU count to account for varying B-cell counts in patients.

In addition, the number of SARS-CoV-2 specific IFN-γ and IL-2 secreting T-cells following polyclonal or SARS-CoV-2 specific stimuli was also determined in a subset of patients using a FluoroSpot kit (FluoroSpot Path: Human IFN-γ/IL-2, SARS-CoV-2, S+NMO, Mabtech AB, Sweden) according to the manufacturer’s instructions. Briefly, in plates pre-coated with monoclonal antibodies (mAbs) specific to human IFN-γ and IL-2, a total of 100,000 PBMCs/well were activated with an anti-CD3 mAb as well as an anti-CD28 mAb (for polyclonal activation). In separate wells, 350,000 PBMCs were instead added into each well and stimulated with anti-CD28 mAb as well as 2μg/mL of SARS-CoV-2 peptides, followed by incubation 20 hr at 37°C and humidified 5% CO_2_ atmosphere. A Mabtech IRIS ELISpot/FluoroSpot reader (Mabtech AB, Nacka Strand, Sweden) was used for the quantification of spot-forming B and T cells (SFCs). Data analysis was carried out with the Mabtech Apex software (version 2.0, Mabtech AB, Nacka Strand, Sweden).

### Statistical analysis

Demographic data were statistically assessed with the use of Fisher’s exact test. For statistical comparisons of total IgG, MFI, flow cytometry and FluoroSpot data, Kruskal-Wallis tests with Dunn’s multiple comparison were performed for comparisons between three groups, and Mann-Whitney tests were performed for comparisons between two groups. Simple linear regressions were carried out to assess correlations between age and total Ig levels and IgG MFI levels in patients. Data regarding nAbs were statistically analyzed with Fisher’s exact tests for categorical variables and Mann-Whitney tests for data with continuous variables. For FACS data, outliers were identified through the ROUT method (28) with a strict Q = 0.1%, where identified outliers were excluded from the analysis. Statistical significance was defined as p-value ≤ 0.05. All statistical analyses were performed using GraphPad Prism (version 10.4.0, Boston, MA, USA).

## Results

### Demography of the study cohort

When comparing SARS-CoV-2 IgG+ patients (n = 78) with the IgG- patients (n = 57), there were no significant differences regarding sex, current level of immunosuppression, or diagnosis (**Table 1**). Six patients received intravenous IgG (IvIG) replacement therapy within six months before sampling of which four children were SARS-CoV-2 IgG+. Most patients (five out of six) on IvIG had a medical history of allo-HSCT. However, significant differences were found with regards to age, where IgG+ patients and siblings, all IgG+, had a significantly higher median age than IgG- patients (p < 0.01). Previous COVID-19 vaccination also differed, where 17.9% of IgG+ patients had been vaccinated compared to only 1.8% vaccinated in IgG- patients (p < 0.001). Within this pediatric oncological and hematological cohort, neither immunosuppression nor cancer diagnosis influenced a patient’s ability to seroconvert in response to SARS-CoV-2.

**Table 1:** Clinical characteristics in patients and healthy siblings.

Clinical characteristics	SARS-CoV-2 IgG- patients (n = 57)	SARS-CoV-2 IgG+ patients (n = 78)	Siblings (n = 14)	p-value
Age in years, median (range)	6 (0–18)	10 (1–17)	13 (2–15)	< 0.01
Age groups				< 0.05
0–3 years old (n = 36)	20 (35.1%)	15 (19.2%)	1 (7.1%)	
4–10 years old (n = 52)	23 (40.4%)	25 (32.1%)	4 (28.6%)	
11–18 years old (n = 61)	14 (24.6%)	38 (48.7%)	9 (64.3%)	
Sex				ns
Boy (n = 74)	26 (45.4%)	40 (51.3%)	8 (57.1%)	
Girl (n = 75)	31 (54.4%)	38 (48.7%)	6 (42.9%)	
Diagnosis^#^				ns
Lymphoma (n = 13)	5 (8.8%)	8 (10.3%)	–	
Leukemia (n = 45)	15 (26.3%)	30 (38.5%)	–	
Solid tumor (n = 39)	18 (31.6%)	21 (26.9%)	–	
CNS tumor (n = 21)	14 (24.6%)	7 (9.0%)	–	
Non-malignant disorder (n = 17)	5 (8.8%)	12 (15.4%)	–	
Immunosuppression				ns
None (n = 22)	9 (15.8%)	13 (16.7%)	–	
Mild (n = 34)	19 (33.3%)	15 (19.2%)	–	
Moderate (n = 28)	12 (21.1%)	16 (20.5%)	–	
Severe (n = 51)	17 (39.8%)	34 (43.6%)	–	
IvIG replacement therapy	1 (1.8%)	5(6.4%)		ns
COVID-19 vaccinated				< 0.001
Yes (n = 22)	1 (1.8%)	14 (17.9%)	7 (50.0%)	
No (n = 116)	51 (89.5%)	58 (74.4%)	7 (50.0%)	
Unknown (n = 11)	5 (8.8%)	6 (7.7%)	0 (0.0%)	

A Kruskal-Wallis test was performed for the continuous variable and Fisher’s exact tests were performed for all categorical variables. Statistically significant p-values ≤ 0.05 are indicated in bold

-, negative; +, positive; CNS, central nervous system; COVID-19, coronavirus disease 2019; IvIG, intravenous immunoglobulin; ns, not significant; SARS-CoV-2, severe acute respiratory syndrome coronavirus 2.

# A detailed description of diagnoses is found in **Supplementary Table 1**.

### Total immunoglobulin levels reflect the degree of immunosuppression

**Figure 2:**
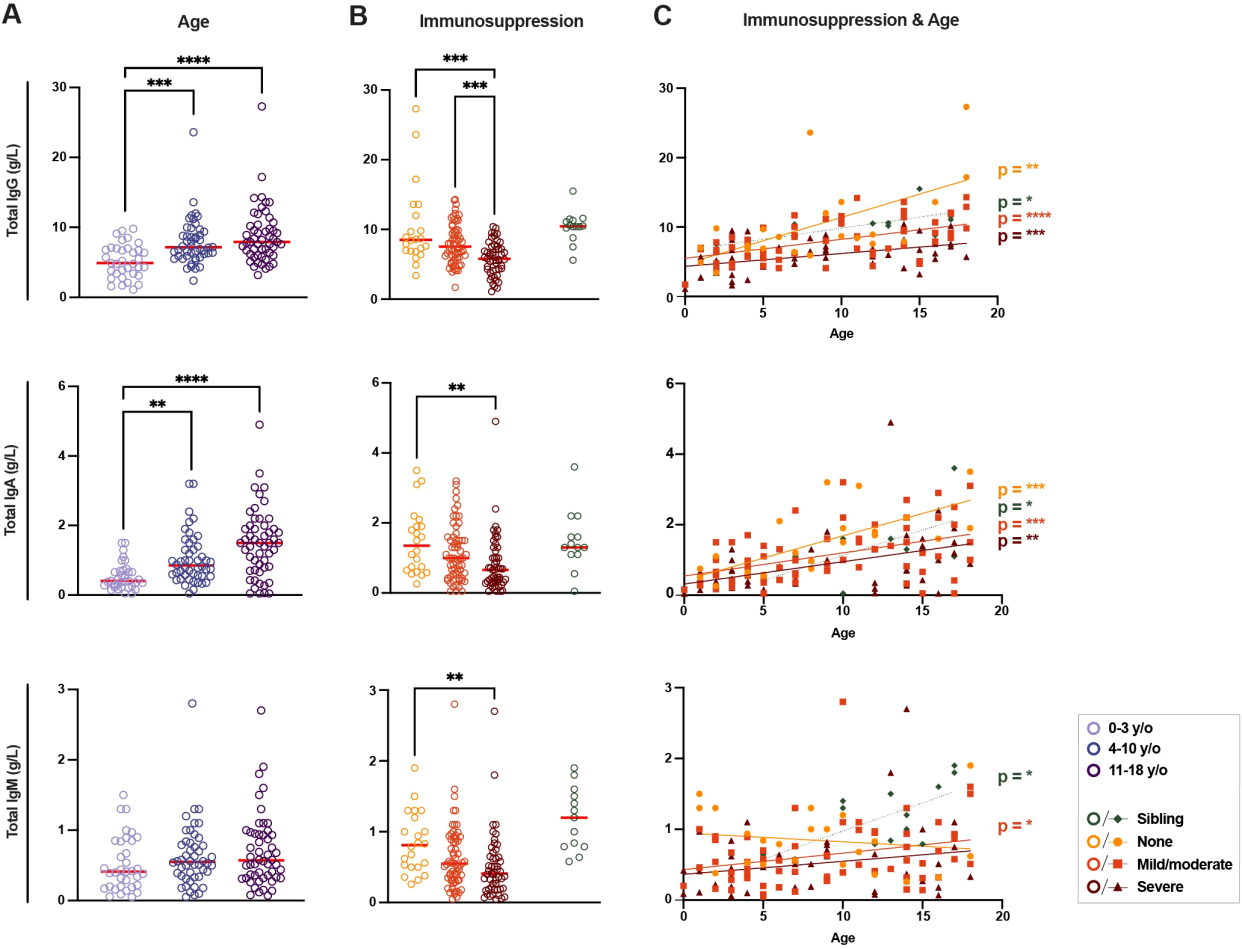
Differences in total immunoglobulin in immunosuppressed children and healthy siblings. **(A)** Levels of total immunoglobulin (Ig) G, IgA, and IgM (g/L) between groups of patients according to age (n = 135) and **(B)** current immunosuppressive treatment (n = 149, including healthy siblings (n = 14)). **(C)** Linear regressions of total IgG, IgA, and IgM levels according to age for all three levels of immunosuppressive treatment and healthy siblings (n = 149). For IgG: p < 0.01 and R^2^ = 0.36 for patients with no immunosuppression, p < 0.0001 and R^2^ = 0.27 for patients with mild/moderate immunosuppression, p < 0.001 and R^2^ = 0.20 for patients with severe immunosuppression and p < 0.05 and R^2^ = 0.42 for siblings; for IgA: p < 0.001 and R^2^ = 0.50 for patients with no immunosuppression, p < 0.001 and R^2^ = 0.20 for mild/moderate, p < 0.01 and R^2^ = 0.20 for severe and p < 0.05 and R^2^ = 0.37 for siblings; for IgM: p < 0.05 and R^2^ = 0.08 for patients with mild/moderate immunosuppression and p < 0.05 and R^2^ = 0.44 for siblings. A Kruskal-Wallis test with Dunn’s multiple comparison test was performed to determine significant differences in age and immunosuppressive groups. Simple linear regressions were run for graphs with total Ig levels plotted against age as a continuous fact. Statistical significance was defined as p ≤ 0.05 (** p ≤ 0.01, *** p ≤ 0.001, **** p ≤ 0.0001) with medians shown as red horizontal lines.

Total (i.e., not SARS-CoV-2-specific) IgG, IgA, and IgM levels were determined and analyzed in all patients to give an overview of the B-cell compartment and to validate the clinically assessed degree of immunosuppression (**Figure 2**). With regards to age, there was a significant trend of higher total IgG levels in older children within the patient cohort (median of 4.90 g/L for 0–3-year-olds vs 7.15 g/L for 4–10-year-olds, p < 0.001 and vs 7.90 g/L for 11–18-year-olds, p < 0.0001, **Figure 2A**). A similar trend was seen with regards to total IgA levels where significantly higher concentrations were observed in 4–10-year-olds and 11–18-year-olds compared to toddlers of 0–3 years of age (median of 0.41 g/L for 0–3-year-olds vs 0.85 g/L for 4–10-year-olds, p < 0.01 and vs 1.50 g/L for 11–18-year-olds, p < 0.0001, **Figure 2A**). No differences were seen in total IgM levels with age. Diagnosis did not impact on immunoglobulin levels. Children substituted with IvIG had normal IgG values according to age (data not shown).

With regards to the varying degrees of immunosuppression in the patient cohort (**Figure 2B**), total IgG levels were significantly lower in patients with ongoing severe immunosuppression compared both to patients without ongoing immunosuppression and patients with only mild/moderate immunosuppression (median of 5.80 g/L in patients with severe immunosuppression vs median of 8.50 g/L in patients with no immunosuppression, p < 0.001, and vs 7.55 g/L with mild/moderate immunosuppression, p < 0.001, **Figure 2B**). Once again, a similar trend was seen when looking at total IgA levels, where patients with severe immunosuppression had significantly lower total IgA concentrations when compared to patients without ongoing immunosuppression (median of 0.66 g/L vs 1.35 g/L, p < 0.01). Finally, with regards to IgM, severely immunosuppressed patients once again had lower levels of IgM compared to non-immunosuppressed patients (median of 0.41 g/L vs 0.81 g/L, p < 0.01). Healthy siblings showed similar levels of total IgA and IgG, and slightly higher IgM levels, compared to patients without ongoing immunosuppression (**Figure 2B**). The differences in total immunoglobulin levels between the groups confirmed the clinically assessed severity of immunosuppression following treatment and its effect on the B-cell compartment.

Analyzing immunosuppression and age together, significant correlations were found in total IgG and IgA levels with age in all immunosuppression groups (**Figure 2C**). The total IgM levels in patients with no immunosuppression showed a negative, though non-significant, trend with age. Siblings and patients with mild/moderate immunosuppression had significant positive correlations between age and IgM levels (**Figure 2C**). The impact of age on total immunoglobulin levels, though affected by immunosuppression, was seen in all patient groups.

### Quality of antibody responses to SARS-CoV-2 in children with cancer or hematologic disease

**Figure 3:**
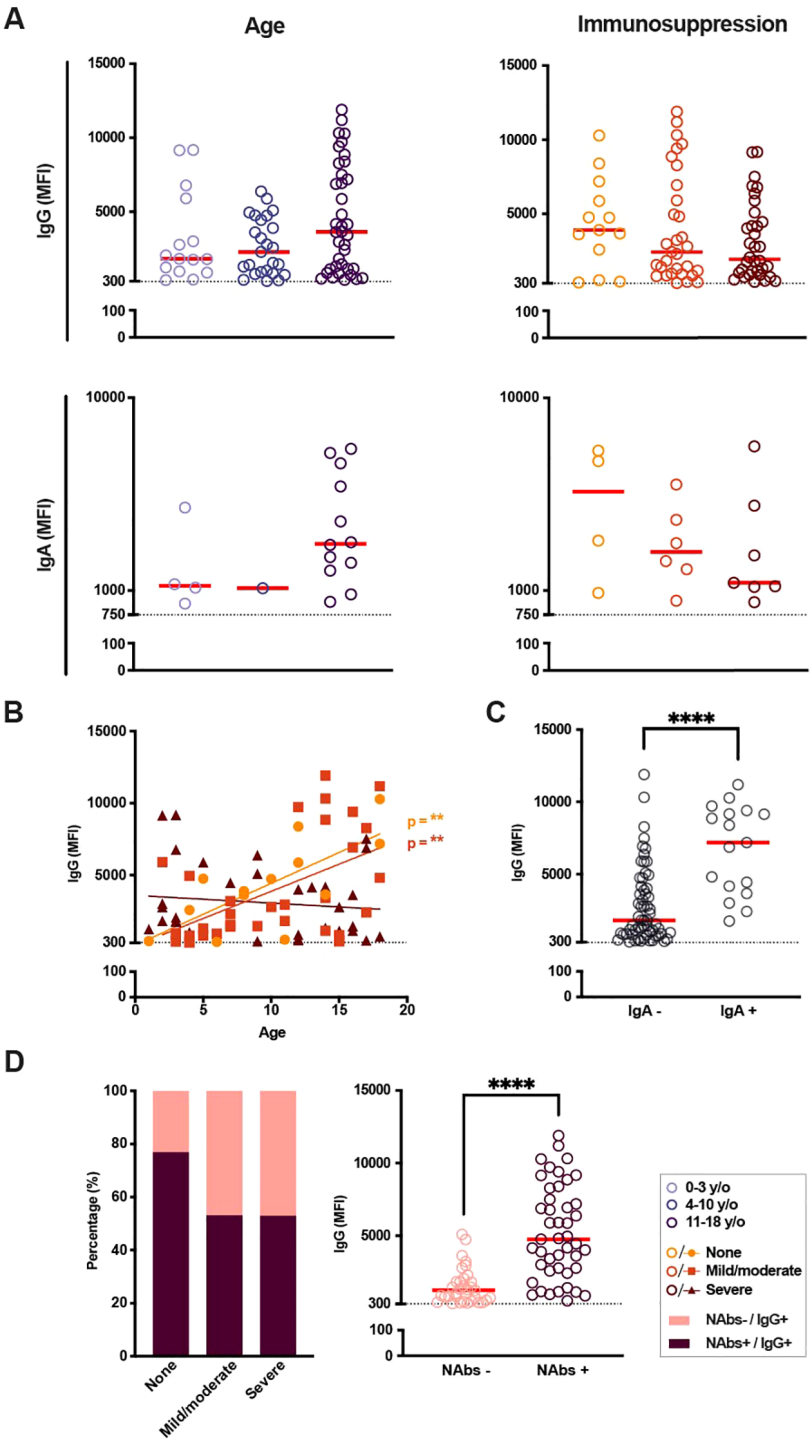
SARS-CoV-2 immunoglobulin levels and presence of neutralizing antibodies in seropositive patients according to age and immunosuppressive treatment. **(A)** SARS-CoV-2-specific immunoglobulin (Ig) G and IgA median fluorescence intensity (MFI) in IgG-seropositive patients based on age and current immunosuppressive treatment. Cut-off (dotted line) for IgG seropositivity set to 300 MFI (n = 78), cut-off (dotted line) for IgA seropositivity to 750 MFI (n = 17). **(B)** IgG MFI with regards to age as a continuous factor in seropositive patients of all immunosuppressive groups (n = 78), p < 0.01 and R^2^ = 0.52 for patients with no immunosuppression, p < 0.01 and R^2^ = 0.28 for patients with mild/moderate immunosuppression. **(C)** IgG MFI depending on IgA seroconversion (n = 78). **(D)** Presence of neutralizing antibodies (nAbs) based on current immunosuppression (none, n = 13; mild/moderate, n = 32; severe, n = 34; left graph) as well as IgG MFI depending on the presence of nAbs (right graph). A Kruskal-Wallis test with Dunn’s multiple comparison test was performed to determine significant differences in age and immunosuppressive groups. A Mann-Whitney test was carried out for significances in IgG MFI based on IgA seropositivity and presence of nAbs. Simple linear regressions were run for graphs with total Ig levels plotted against age as a continuous fact. Statistical significance was defined as p ≤ 0.05 (**** p ≤ 0.0001) with medians shown as red horizontal lines.

SARS-CoV-2-specific antibodies were qualitatively evaluated in the 78 SARS-CoV-2 IgG+ patients and 14 siblings (**Figure 3**). No significant variations in SARS-CoV-2-specific IgG MFI levels were found in SARS-CoV-2 IgG+ patients with regards to immunosuppression or age (**Figure 3A**, upper panel). Clinical diagnosis did not impact on the SARS-CoV-2 antibody responses (data not shown). However, weak trends could be noticed, with increasing SARS-CoV-2-specific IgG MFI with age and decreasing MFI with increasing immunosuppression. In addition, SARS-CoV-2 IgG MFI showed a weak correlation to total IgG (**Supplementary Figures 1A, B**).These trends were also observed when quantifying through ELISA, which correlated well with MFI levels, though, once again, no significant differences were found (**Supplementary Figures 1C, D**). SARS-CoV-2 IgA seroconversion occurred in fewer patients (n = 17) and showed similar trends of lower SARS-CoV-2-specific IgA MFI levels with increasing immunosuppression and slightly higher IgA MFI levels with age, though these differences were not statistically significant (**Figure 3A**, lower panel). Analyzing age and immunosuppression together, a significant correlation between age and SARS-CoV-2-specific IgG MFI was found in patients with no and mild/moderate immunosuppression but was non-existent in patients with severe immunosuppression (**Figure 3B**). Finally, it was observed that an important factor for IgG MFI levels was whether IgA seroconversion occurred or not (**Figure 3C**), with IgA+ patients showing significantly higher IgG MFI levels compared to IgA- patients (median of 7,186 MFI vs 1,815 MFI, p < 0.0001).

The quality of the SARS-CoV-2 antibody responses was further assessed through the determination of SARS-CoV-2 nAbs in serum. Most importantly, SARS-CoV-2 nAbs were found in patients of all ages and degrees of immunosuppression. When comparing the proportions of IgG+ patients with or without nAbs, grouped by immunosuppression, we found a slightly larger occurrence of nAbs in patients with no current immunosuppression compared to patients with mild/moderate or severe immunosuppressive treatment (76.9% vs 53.1% and 52.9%, respectively). The presence of nAbs was significantly correlated with the presence of SARS-CoV-2 IgA response, as well as increased IgG and IgA MFI levels (p < 0.0001, respectively **Figure 3D**; **Table 2**).

**Table 2:** Clinical characteristics for patients according to presence of neutralizing antibodies.

Clinical characteristics	nAbs- (n = 33)	nAbs+ (n = 45)	p-value
Age in years, median (range)	7 (1–18)	12 (2–18)	ns
Age groups			< 0.05
0–3 years **old (n = 15)**	7 (21.2%)	8 (17.8%)	
4–10 years **old (n = 25)**	15 (45.5%)	10 (22.2%)	
11–18 years **old (n = 38)**	11 (33.3%)	27 (60.0%)	
Sex			ns
Boy (n = 40)	14 (42.4%)	26 (57.8%)	
Girl (n = 38)	20 (57.6%)	19 (42.2%)	
Diagnosis^#^			ns
Lymphoma (n = 8)	5 (15.2%)	3 (6.7%)	
Leukemia (n = 30)	15 (45.5%)	15 (33.3%)	
Solid tumor (n = 21)	8 (24.2%)	13 (28.9%)	
CNS tumor (n = 7)	1 (3.0%)	6 (13.3%)	
Non-malignant (n = 12)	4 (12.1%)	8 (17.8%)	
Allo-HSCT			ns
Yes (n = 14)	5 (15.2%)	9 (20.0%)	
No (n = 64)	28 (84.4%)	36 (80.0%)	
Immunosuppression			ns
None (n = 13)	3 (9.1%)	10 (22.2%)	
Mild (n = 15)	8 (24.2%)	7 (15.6%)	
Moderate (n = 16)	6 (18.2%)	10 (22.2%)	
Severe (n = 34)	16 (48.5%)	18 (40.0%)	
Total Ig, median (range)			
Total IgG g/L	6.9 (2.4–14.2)	7.3 (2.1–27.3)	ns
Total IgA g/L	0.72 (0.05–3.20)	1.00 (0.05–4.90)	ns
Total IgM g/L	0.36 (0.07–1.50)	0.62 (0.05–2.70)	**< 0.05**
COVID-19 vaccination			ns
Yes (n = 14)	4 (12.1%)	10 (22.2%)	
No (n = 58)	25 (75.8%)	33 (73.3%)	
Unknown (n = 6)	4 (12.1%)	2 (4.4%)	
SARS-CoV-2 IgA response			< 0.0001
IgA- (n = 61)	33 (100.0%)	28 (62.2%)	
IgA+ (n = 17)	0 (0.0%)	17 (37.8%)	
SARS-CoV-2 MFI median (range)			
IgG MFI	1,242 (331–5,096)	4,757 (516–11,904)	**< 0.0001**
IgA MFI	73 (0–421)	542 (0–5,436)	**< 0.0001**

Mann-Whitney tests were performed for all continuous variables and Fisher’s exact tests for all categorical variables. Statistically significant p-values ≤ 0.05 are indicated in bold.

Allo-HSCT, allogeneic hematological stem cell transplant; CNS, central nervous system; COVID-19, coronavirus disease 2019; IgA, immunoglobulin A; IgG, immunoglobulin G; IgM, immunoglobulin M; MFI, median fluorescence intensity; nAbs, neutralizing antibodies; ns, not significant; -, negative; +, positive; SARS-CoV-2, severe acute respiratory syndrome coronavirus 2.

# A detailed description of diagnoses is found in **Supplementary Table 1**.

Thereafter, an attempt was made to elucidate possible factors correlating with the presence of SARS-CoV-2 nAbs in this cohort (**Table 2**). There was a significant correlation with age, where the proportion of teenagers was higher in patients with nAbs compared to patients without (p < 0.05). Neither sex, immunosuppression, diagnosis, previous allo-HSCT, nor vaccination correlated with the presence of nAbs. On the other hand, nAbs was significantly correlated with higher MFI levels of IgG (p < 0.0001) and IgA (p < 0.0001) as well as SARS-CoV-2 IgA seroconversion (p < 0.0001). Finally, total IgM levels were also higher in nAbs+ patients (p < 0.05). Regardless of immunosuppression and immunoglobulin levels, age was found to be a greater determinant of the patients’ ability to effectively respond to a SARS-CoV-2 infection in terms of IgG and IgA responses and neutralizing capacity.

### Memory B-cell responses to SARS-CoV-2 proteins

**Figure 4:**
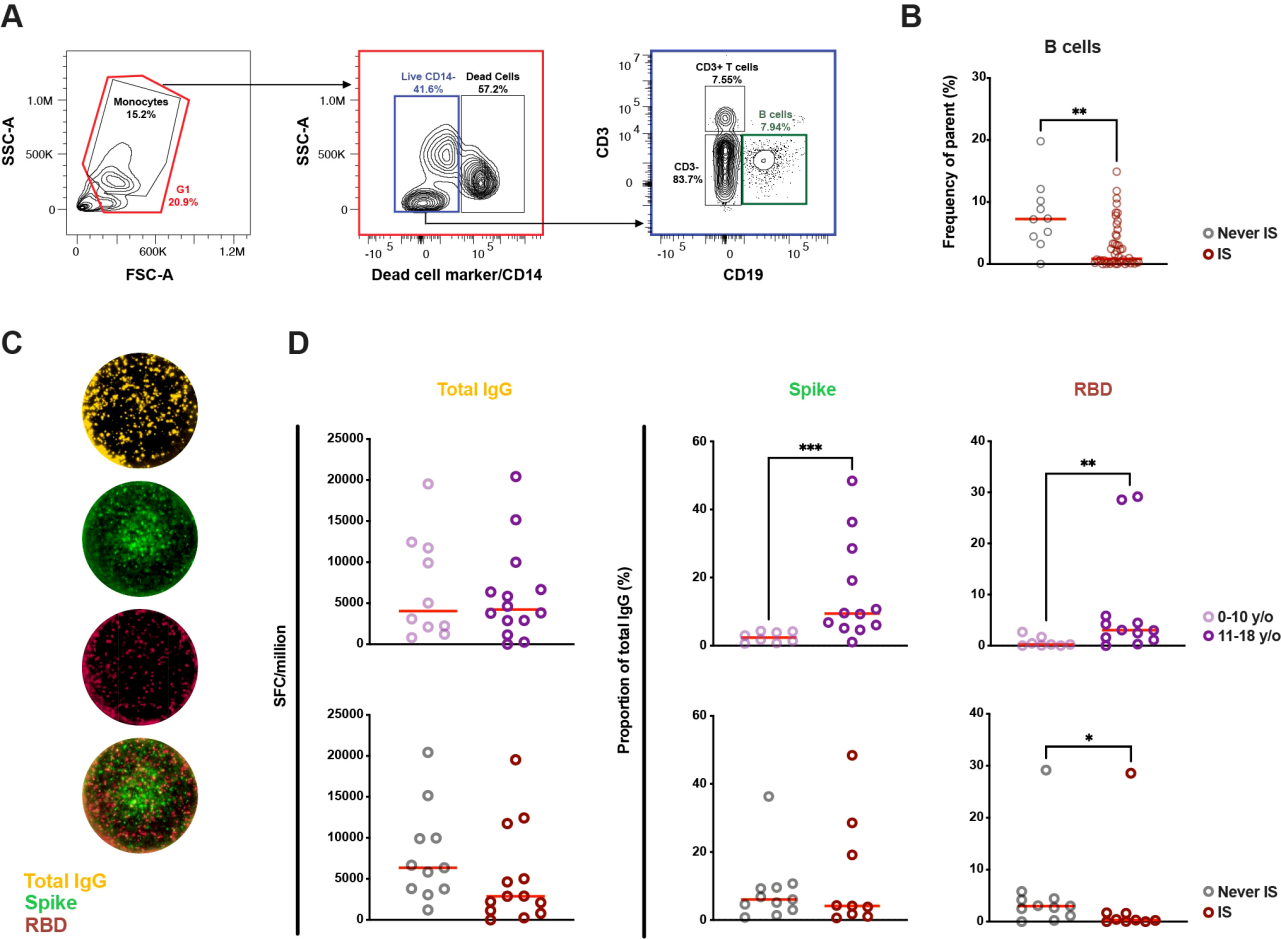
Difference in B-cell proportions and memory B-cell responses in children based on age and previous or ongoing immunosuppressive treatment in life. **(A)** Representative gating strategy of the B-cell population from peripheral blood mononuclear cells. **(B)** Proportion of B cells in children exposed to immunosuppression or never exposed (n = 56). In five patients, PBMCs from a subsequent time-point was used for the Fluorospot assay compared to serology. **(C)** Representative FluoroSpot wells from a 12-year-old patient with Diamond-Blackfans anemia showing total IgG (yellow) as well as IgG specific to spike (S) (green) and receptor-binding domain (RBD) (red) of SARS-CoV-2 following pre-stimuli with IL-2 and R848. **(D)** Count of total IgG spot forming cells (SFC) as well as proportion of RBD and S SFCs depending on age and previous or ongoing immunosuppression in life (n = 24 for total IgG, n = 20 for RBD and S). A Mann-Whitney test was performed to determine significant. Statistical significance was defined as p ≤ 0.05 (* ≤ 0.05, ** ≤ 0.01, *** p ≤ 0.001) with medians shown as red horizontal lines. IS, immunosuppression; SFU, spot-forming units.

Although the B-cell compartment in severely immunosuppressed patients exhibited a general suppressive state, as indicated by total immunoglobulin levels, this suppression was not reflected in the levels or functionality of SARS-CoV-2-specific antibodies. To further investigate the B-cell compartment, PBMCs from both patients and their siblings were immunophenotyped, using flow cytometry. (**Figure 4A**). Due to the low number of PBMC samples available, both patients and siblings were from now on grouped by and compared based on age (0–10 years vs 11–18 years of age) and whether they ever experienced immunosuppressive treatment or not. As expected, the frequency of B-cells (live CD14^-^/CD19^+^ single cells) was significantly lower in children exposed to immunosuppression compared to children who had never been exposed to immunosuppression (median of 0.87% vs 7.29%, p < 0.01, **Figure 4B**).

Following this, an attempt was made to bring further clarity to cellular B-cell memory against SARS-CoV-2 through FluoroSpot (**Figure 4C**). As the flow cytometric analysis showed significant differences in B-cell proportions between children with and without immunosuppression, RBD- and S-specific IgG SFCs were normalized to total IgG SFCs to account for differences in B-cell proportions. Total IgG SFCs did not differ between young children (0–10-year-olds) compared to older children (11–18-year-olds) (**Figure 4D**). However, age correlated to the proportion of S-specific IgG SFCs, with a significantly higher median in older children compared to younger children (median of 9.43% vs 2.40%, p < 0.001). A similar difference was also observed in RBD-specific IgG SFCs (median of 3.03% vs 0.24%, p < 0.01). With regards to immunosuppression, there were no significant differences in total IgG SFCs between children with or without experience of immunosuppression (**Figure 4D**), nor were there any significant differences seen when comparing the proportions of S-specific IgG SFCs. However, children who had experienced immunosuppression showed a significantly lower proportion of RBD-specific IgG SFCs compared to those who had not (median of 3.01% vs 0.31%, p < 0.05) despite having similar age between the groups (data not shown). These results imply that age has the largest effect on specific MBCs to SARS-CoV-2 S- and RBD-specific IgG in general, while immunosuppression negatively impacts the differences seen in RBD-specific IgG-secreting cells.

### Association between polyclonal and SARS-CoV-2 specific IFN-γ and IL-2 producing T-cells

Fourteen patients with IgG antibodies against SARS-CoV-2 had enough PBMCs for T-cell FluoroSpot analysis as well. Representative images of responses following T-cell activation at bulk (with anti-CD3) or at antigen-specific level (SARS-CoV-2 peptides) are depicted in **Supplementary Figure 2A**. Overall, activation at bulk T-cell level with antigen elicited in general higher numbers of IFN-γ and IL-2 responses compared to polyfunctional IFN-γ and IL-2 responses (**Supplementary Figure 2B**). As sample size was limited, no statistical significances were calculated. However, there seems to be a trend towards lower IFN-γ and especially IL-2 responses to SARS-CoV-2 peptides with more severe immunosuppression.

## Discussion

The COVID-19 pandemic brought many challenges to children with cancer or hematological disease worldwide. However, it also allowed for the study of immune responses to a completely new viral pathogen in immunocompromised patients. This single-center cohort was rapidly set up in June 2020 with the overall aim to bring insights into how infection with SARS-CoV-2 affects immunosuppressed children. Previous findings from this cohort and others have shown that most children with cancer or hematological diseases have mild symptoms of COVID-19 and that antibodies against SARS-CoV-2 can be detected in patients upon infection (7, 29, 30). The present study brings greater depth into our understanding of the quantity and quality of B-cell responses to SARS-CoV-2 in immunosuppressed children.

First, we analyzed total immunoglobulin levels to mirror the assessed immunosuppression state, regarding both age and treatment intensity. Our findings align with previous pediatric research supporting the idea that immunoglobulin levels increase with age and immune maturation (16, 31) as well as with previous findings of lower immunoglobulin levels in children undergoing cancer treatment (32, 33). Despite the lower total immunoglobulin levels observed with increasing immunosuppression, children with ongoing severe immunosuppression were able to seroconvert against SARS-CoV-2 with antigen-specific IgG and IgA. Our data shows a non-significant trend of increasing SARS-CoV-2 S1-specific IgG and IgA MFI levels with age and, on the other hand, decreasing levels with increased immunosuppression. In patients with no or mild/moderate immunosuppression, IgG MFI levels increased with older age, a trend that we did not observe in patients with severe immunosuppression. It is conceivable that a high degree of treatment intensity causing severe immunosuppression may impair antibody production upon natural infection.

To our knowledge, despite their correlation with protective immunity (34), the development of nAbs against SARS-CoV-2 in pediatric oncological and hematological patients has previously mostly been studied in vaccine contexts (19, 21, 35). Within our study, most patients were not vaccinated, as children with cancer were not included in the Swedish vaccine recommendations during the pandemic unless they had undergone allo-HSCT in the past. Of note, our data show that children with cancer were able to develop nAbs regardless of diagnosis or immunosuppression, with the main determining factor being older age. Whether this reflects an immunological difference with increasing age or whether the older patients in our cohort had suffered repeat infections or symptomatic infections is not fully elucidated. Furthermore, nAbs+ patients exhibited higher IgG MFI levels, a finding consistent with previous studies showing that elevated SARS-CoV-2 IgG levels correlated to neutralizing activity in both adults and healthy children (36, 37). Additionally, in our study IgA seroconversion was also correlated with the presence of nAbs, which has previously been observed in adults without cancer (36, 38). It is important to note that whilst IgA was only measured in serum in this study, previous research has demonstrated that IgA responses in serum are strongly correlated to levels in saliva (39). Furthermore, the presence of nAbs was determined by a reduction in plaque formation during the PRNT, which just measures the functional humoral part of the immune response, i.e., we did not investigate innate or cellular immune responses (17). Despite these limitations, our findings suggest that increasing age is associated with a broader antibody response, characterized by higher SARS-CoV-2 IgG MFI levels, IgA seroconversion, and the presence of nAbs, even in immunosuppressed children. Our findings corroborate previous studies showing loss of protective antibodies for vaccine antigens encountered after cancer treatment (9). Also here, young age is shown to be a risk factor for loss of protective antibodies (40) while older children more often display preserved vaccine antibody titers after chemotherapy (11, 41). In the clinical setting, our findings suggest that young children may benefit from tailored revaccination strategies as well as close monitoring of IgG and intravenous replacement therapy in case of hypogammaglobulinemia (42).

To clarify the role of SARS-CoV-2 B-cell immunity, we evaluated antigen-specific MBC responses, focusing on those generated through the germinal center (GC) reaction. When stratified by age or immunosuppression status, there were no significant differences in the total number of IgG SFCs between the groups. However, SARS-CoV-2-specific responses varied significantly across age groups, with older individuals showing increased numbers of both S and RBD IgG SFCs, a finding that aligns with previous data in healthy children (43). Interestingly, in our cohort of children, immunosuppression was associated with a significant reduction in the number of RBD IgG SFCs, but not S IgG SFCs. We hypothesize that this may be due to a blunted GC reaction caused by immunosuppressive treatment, as has been suggested in immunosuppressed adults (44) where SARS-CoV-2-specific GC B-cell populations were strongly associated with the ability to produce SARS-CoV-2 nAbs. In that perspective, it is interesting that almost half of the SARS-CoV-2 IgG+ patients in our study lacked nAbs to SARS-CoV-2. This contrasts with a study of SARS-CoV-2 mRNA vaccination in children undergoing chemotherapy where only 12% of the vaccinated children lacked SARS-CoV-2 nAbs after three doses of vaccine (21). Upon follow up of the same vaccinated cohort, 20% lacked nAbs over a year later (22). Further studies with a larger sample size are needed to validate these observations and explore any emerging trends. Whether natural infection in children undergoing cancer treatment will result in persistent and efficient MBC responses upon re-infection, as reported for healthy adults (45), remains to be investigated. Nonetheless, these preliminary results suggest that age and immunosuppressive treatment may influence B-cell immunity in distinct ways.

It is important to recognize that humoral immunity alone does not fully account for effective antiviral defense mechanisms. Further research is warranted to delineate the immunological impact of cancer and its treatment in pediatric populations. T-cell studies have demonstrated functional impairments following chemotherapy, which increased with increasing numbers of chemotherapy cycles (46, 47). In our cohort, prior findings indicated that bone marrow-suppressive therapy was associated with reduced numbers of IFN-γ producing T-cells in response to varicella zoster virus, a clinically relevant herpesvirus in the pediatric oncology setting (48). Although the number of patients with available T-cell data is limited in the current study, trends suggest a decrease in IFN-γ and IL-2 producing T-cells specific to SARS-CoV-2 peptides in patients with ongoing immunosuppression which should be further examined in a larger population. Collectively, it appears that pediatric cancer patients may experience not only compromised humoral responses but also broader deficits in adaptive immunity, potentially requiring extended periods for immunological recovery. The heterogeneity of our cohort is both a strength and a limitation. Pediatric cancers are rare, but thanks to the width of diagnoses and treatments present within this cohort, our results could contribute to knowledge and conclusions regarding the whole pediatric oncology and hematology spectrum. A clear limitation, however, lies in the unknown history of SARS-CoV-2 infection in most patients, as PCR testing was solely performed based on symptoms resulting in few confirmed cases (data not presented). Nevertheless, it should be stated that previous pediatric studies have shown that humoral immunity against SARS-CoV-2 is preserved for months to years (2, 37, 49, 50). Also, as the pandemic developed and the SARS-CoV-2 virus evolved rapidly during the study period, study participants were exposed to different SARS-CoV-2 variants, which may lead to differing immune responses, notably due to the immune escaping capabilities of the Omicron variant. However, by analyzing our samples using wild-type, Delta, and Omicron antigens, the wild-type antigen was deemed appropriate to use for analysis. Finally, while most study participants were not vaccinated against SARS-CoV-2, we lack data on the number of (re-)infections and immunizations, which may affect the quality and quantity of the immune responses. This may be especially true for SARS-CoV-2 vaccinated cases, as studies of pediatric cancer patients suggest that vaccination is superior to natural infection in generating a robust humoral immune response including nAbs responses (22, 51). Finally, all patients included in our study experienced asymptomatic or mild SARS-CoV-2 natural infection reflecting the very few severe SARS-CoV-2 cases in children with cancer in Sweden. As studies in adults after COVID-19 have failed to show any differences in memory B- and T-cell responses depending on disease severity (52, 53), we do not believe this skews our data.

## Conclusion

Our results show that most pediatric oncological and hematological patients can mount a broad antibody response upon SARS-CoV-2 natural infection or vaccination, although there is a variability in their response mostly influenced by increasing age. MBC responses in children with immunosuppression were blunted with fewer RBD IgG-secreting cells. Essentially, our findings underscore that young children with severe treatment-related immunosuppression are at risk for less effective B-cell responses upon viral infection. This is in line with previous studies on vaccine B-cell immunity after chemotherapy and should be considered in management of clinical infections and for vaccination strategies in childhood cancer survivors.

## Data Availability

The raw data supporting the conclusions of this article will be made available by the authors, without undue reservation.

## Supplementary material

Supplementary Figure 1 SARS-CoV-2 IgG levels in seropositive children measured through Enzyme-Linked Immunosorbent Assay. **(A)** Variations in SARS-CoV-2 IgG levels in seropositive patients based on age and level of immunosuppression. **(B)** Correlation between quantification of SARS-CoV-2 IgG through Enzyme-Linked Immunosorbent Assay (ELISA) (Spike (S) trimer, units/mL) and suspension multiplex immunoassay (SMIA) (S1, median fluorescence intensity (MFI)). A Kruskal-Wallis test with Dunn’s multiple comparison test was performed to determine significant differences between age and immunosuppressive groups. A correlation was calculated with a nonparametric Spearman correlation (**** p < 0.0001, R = 0.8571). Statistical significance was defined as p ≤ 0.05 with medians shown as red horizontal lines.

Supplementary Figure 2 Levels of IFN-γ and IL-2 secreting T cells in seropositive patients grouped by level of immunosuppression. **(A)** Representative Fluorospot well image for IFN-γ and IL-2 spot-forming units (SFUs) after polyclonal activation (anti-CD3+anti-CD28) (upper row) or antigen-specific activation (SARS-CoV-2 peptides+anti-CD28) (lower row). **(B)** Patients were group into three groups based on immunosuppression, either no ongoing immune suppression (n=3), mild/moderate immunosuppression (n=4) or severe immunosuppression (n=7). No statistical test was carried out.
