# Supplementary figures and images for: The quantity and quality of B-cell immunity against SARS-CoV-2 in children with cancer and hematological diseases
Source: Front Immunol. 2025 Jul 2;16:1613778. doi: 10.3389/fimmu.2025.1613778 (PMC12263943; doi:10.3389/fimmu.2025.1613778)

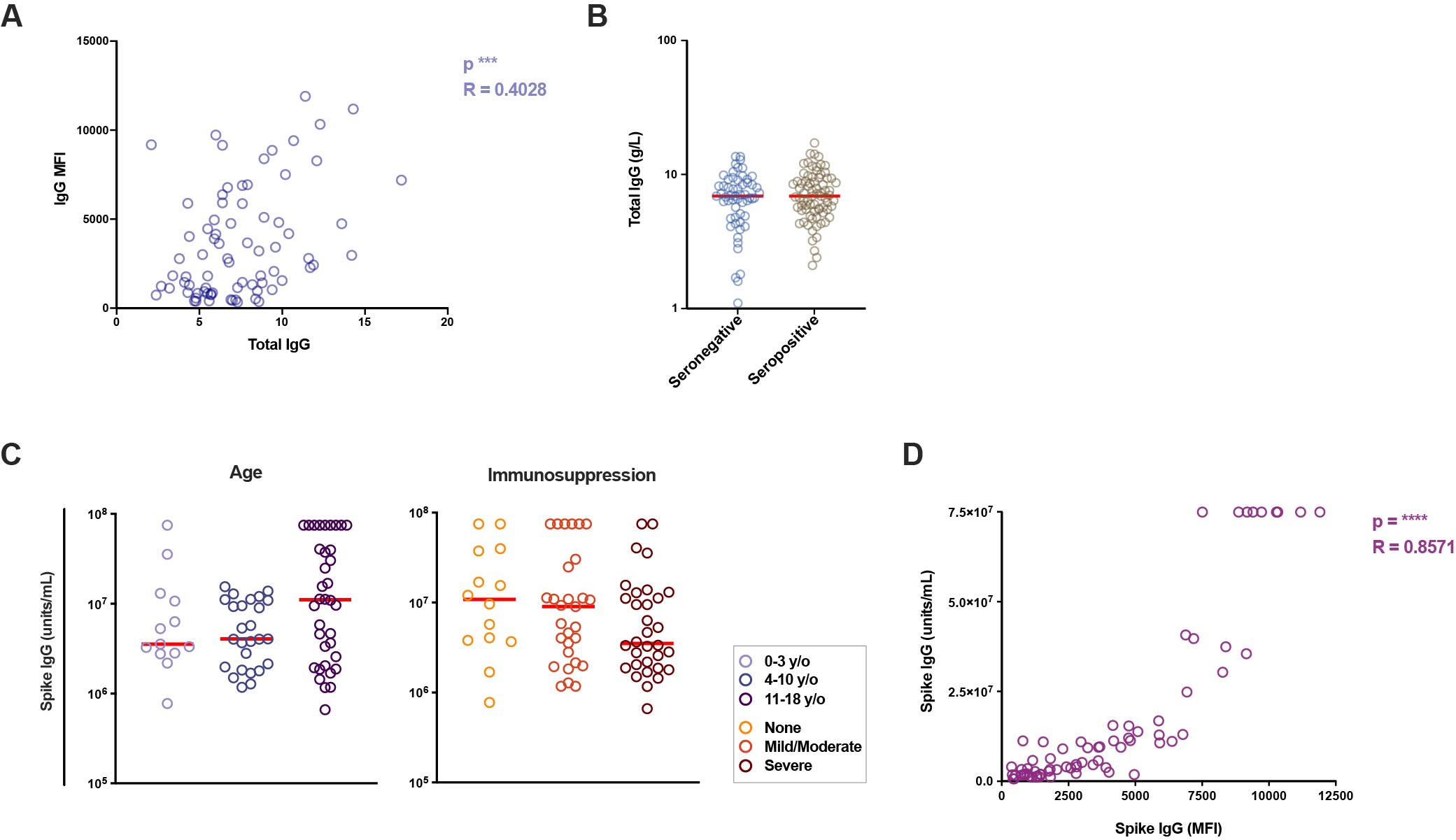

Supplement: Supplementary Figure 1 — SARS-CoV-2 IgG levels in seropositive children measured through Enzyme-Linked Immunosorbent Assay. (A) Variations in SARS-CoV-2 IgG levels in seropositive patients based on age and level of immunosuppression. (B) Correlation between quantification of SARS-CoV-2 IgG through Enzyme-Linked Immunosorbent Assay (ELISA) (Spike (S) trimer, units/mL) and suspension multiplex immunoassay (SMIA) (S1, median fluorescence intensity (MFI)). A Kruskal-Wallis test with Dunn’s multiple comparison test was performed to determine significant differences between age and immunosuppressive groups. A correlation was calculated with a nonparametric Spearman correlation (**** p < 0.0001, R = 0.8571). Statistical significance was defined as p ≤ 0.05 with medians shown as red horizontal lines. [file Image1.jpeg]

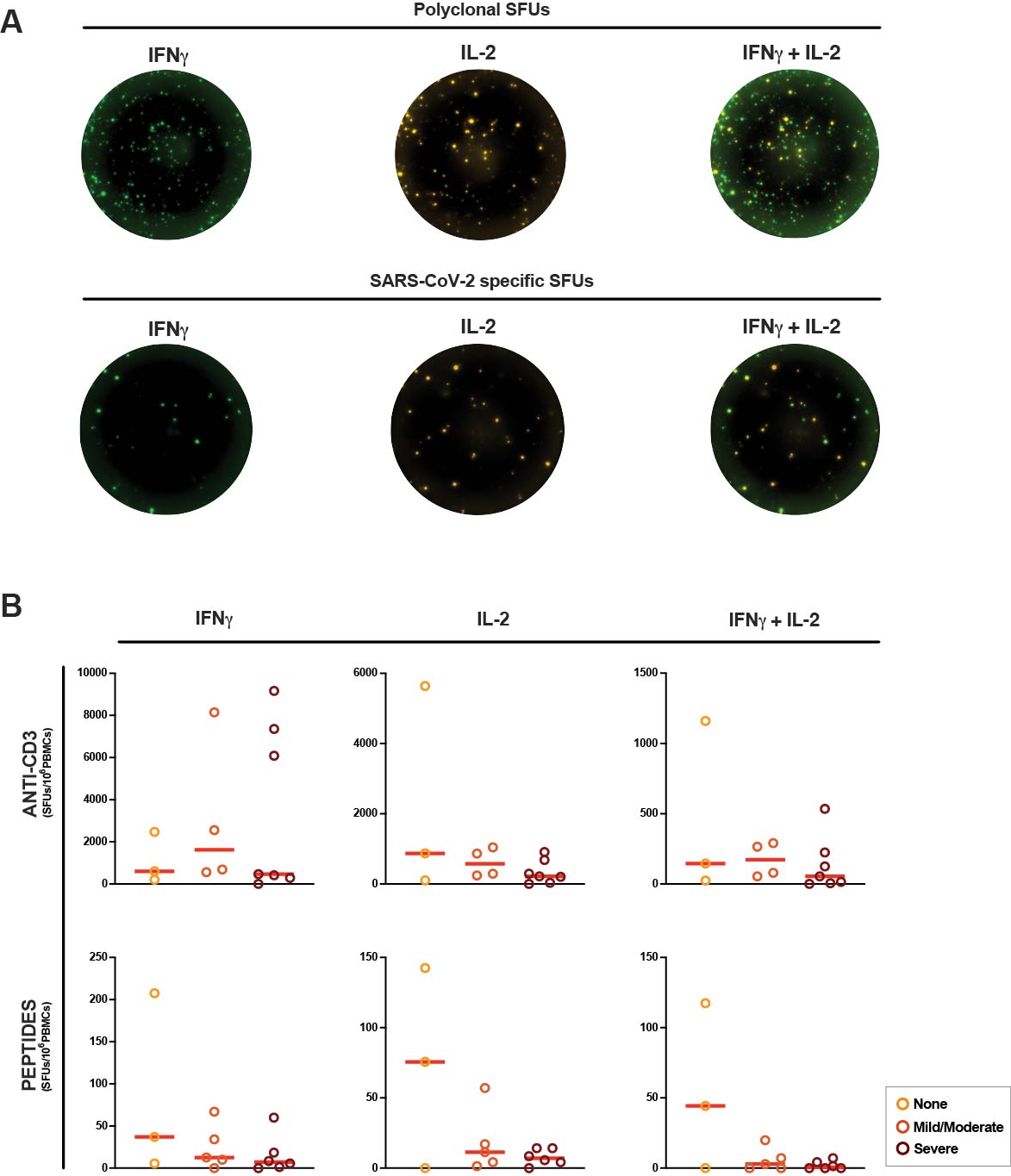

Supplement: Supplementary Figure 2 — Levels of IFN-γ and IL-2 secreting T cells in seropositive patients grouped by level of immunosuppression. (A) Representative Fluorospot well image for IFN-γ and IL-2 spot-forming units (SFUs) after polyclonal activation (anti-CD3+anti-CD28) (upper row) or antigen-specific activation (SARS-CoV-2 peptides+anti-CD28) (lower row). (B) Patients were group into three groups based on immunosuppression, either no ongoing immune suppression (n=3), mild/moderate immunosuppression (n=4) or severe immunosuppression (n=7). No statistical test was carried out. [file Image2.jpeg]
