# Supplementary material for: The quantity and quality of B-cell immunity against SARS-CoV-2 in children with cancer and hematological diseases
Source: Front Immunol. 2025 Jul 2;16:1613778. doi: 10.3389/fimmu.2025.1613778 (PMC12263943; doi:10.3389/fimmu.2025.1613778)
Supplement: Supplementary file 3 [file Table1.docx]

**Supplemental Table 1**: Characteristics of the diagnosis groups included in this study. Rare diseases with only one individual is grouped together as ”Others”.

| **Diagnosis groups** | **Diseases** | **Allo-HSCT** |
| --- | --- | --- |
| Lymphoma (n = 13) | Hodgkin lymphoma (n=8)  Non- Hodgkin lymphoma (n=5) |  |
| Leukemia (n = 45) | ALL (n=33)  AML (n=6  Others (n=6) | (n=9)*  (n=3)  n=1) |
| Solid tumor (n = 39) | Sarcoma (n=12)  Kidney tumor (n=6)  Liver tumour (n=2)  Neuroblastoma (n=11)  Others (n=8) |  |
| CNS tumor (n = 21) | Low-grade tumours (n=5)  High-grade tumors (n=11)  DIPG (n=2)  Other s(n=3) |  |
| \| Non-malignant disorder (n = 17) \| \| --- \| | Thalassemia major (n=5)  Aplastic anemis (n=4)  LCH and HLH (n=2+1)  Others (n=5) | (n=1)  (n=1)  (n=2) |

*One patient received CAR-T cell therapy before allo-HSCT
